# A Case of Complete Heart Block With Diagnostic Challenge and Therapeutic Dilemma

**DOI:** 10.1177/2324709618788110

**Published:** 2018-07-16

**Authors:** Ying Chi Yang, Rama Kanth Pata, Thein Tun Aung

**Affiliations:** 1 Interfaith Medical Center, Brooklyn, NY, USA; 2 University of Iowa Hospitals and Clinics, Iowa City, IA, USA

**Keywords:** asymptomatic, congenital complete heart block, narrow complex escape rhythm, permanent pacemaker implantation

## Abstract

Permanent pacemaker implantation is a class I indication for all symptomatic patients with complete heart block either congenital or acquired. However, certain portions of patients with congenital complete heart block are asymptomatic. Those patients are often very young, and implanting a permanent pacemaker is not always an easy decision. A therapeutic dilemma arises when a select patient population does not meet certain criteria to gain the maximum benefits out of prophylactic pacemaker therapy. Most asymptomatic patients with congenital complete heart block will eventually become symptomatic and require pacemakers at some point in their life but the definitive answer for the ideal time to initiate pacemaker therapy in such population has not been established. We present a case of asymptomatic congenital complete heart block with junctional escape rhythm, which is capable of incrementing the heart rate with physical activity to result in a challenge in diagnosis as well as the treatment strategy.

## Introduction

Third-degree heart block is widely known as complete heart block (CHB). The diagnosis criteria include the presence of complete atrioventricular (AV) dissociation with the atrial rate being higher than the ventricular rate. Majority of patients with CHB are symptomatic due to profound bradycardia. Implanting a permanent pacemaker (PM) is a class I indication for all symptomatic patients with CHB, either congenital or acquired. However, certain portions of patients with congenital complete heart block (CCHB) are asymptomatic. A therapeutic dilemma arises when a select patient population does not meet criteria to gain the maximum benefits out of prophylactic PM therapy. We present a case with asymptomatic congenital CHB with junctional escape rhythm, which is capable of incrementing the heart rate with physical activity to result in a challenge in diagnosis as well as in the treatment strategy.

## Case Presentation

A 23-year-old African American female with no known past medical history presented to the emergency department with 3 days history of nonproductive cough and runny nose. Review of systems was otherwise negative denying chest pain, dizziness, palpitation, or syncope. The patient was not taking any medications. She had no recent travel or positive family history. On physical examination, the patient appeared comfortable. She was afebrile with blood pressure of 107/74 mm Hg, heart rate of 45/minute, and oxygen saturation of 99% on ambient air. The patient had mild pharyngeal edema but no jugular venous distension. Auscultation of the heart revealed slow heart rate, but it was regular with normal first and second heart sounds having no murmurs. Auscultation of bilateral lungs revealed clear breath sounds. There were neither skin rash nor pedal edema.

**Figure 1.**
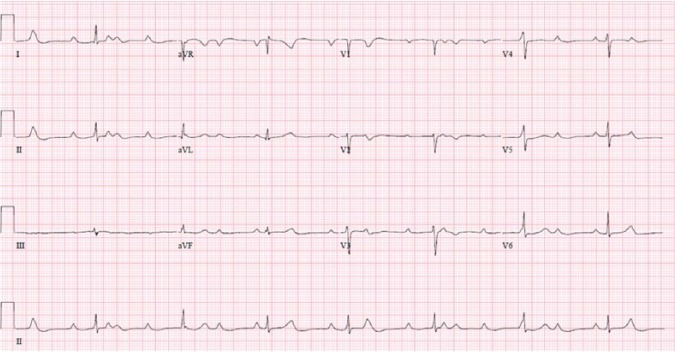
Twelve-lead electrocardiogram on admission showing complete heart block with junctional escape rhythm, atrial rate around 90/minute, and ventricular rate of 45/minute.

Admission electrocardiogram (ECG; Figure 1) showed CHB characterized by AV dissociation with narrow QRS escape rhythm, atrial rate of 90/minute, and ventricular rate of 45/minute. Chest X-ray was unremarkable. Complete blood count and chemistry panel were within normal limits. Troponin, erythrocyte sedimentation rate, and thyroid panel were also within normal limits. Urine toxicology was negative. Lyme IgM antibody, antinuclear antibody, and rheumatoid factor were also negative.

**Figure 2.**
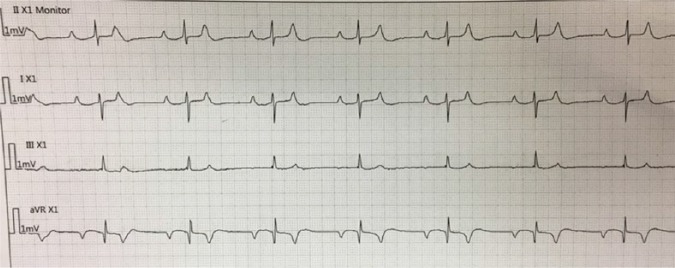
Telemetry rhythm strips showing apparent 2:1 heart block with every other P wave is buried in T wave.

**Figure 3.**
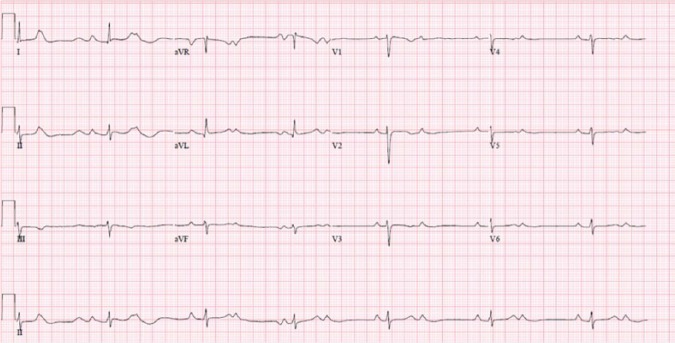
Twelve-lead electrocardiogram showing complete heart block with junctional escape rhythm, isorhythmic atrioventricular dissociation and ventriculophasic sinus arrhythmia, atrial rate around 80/minute, and ventricular rate around 40/minute.

The patient was admitted to the cardiac care unit in the diagnosis of CHB with profound bradycardia at rest. Throughout her hospital stay, the patient remained asymptomatic. She occasionally switched to apparent 2:1 heart block on the telemonitor as shown in Figure 2. Her average systolic blood pressure was around 100 mm Hg, and her average heart rate was 40 to 50 beats per minute. The patient’s heart rate fluctuated along with her physical activity, the lowest being 32/minute during sleep and the highest being 116/minute during exertion. Transthoracic echocardiogram revealed normal left ventricular systolic and diastolic function without major valvular or structural abnormalities. Exercise stress test was performed to assess the patient’s chronotropic competency to physical activity. The patient achieved Bruce protocol stage 3 without any symptoms. She exercised for 10 minutes and achieved metabolic equivalent 12.4. The maximum heart rate during exercise was 139/minute, and she remained in junctional escape rhythm with CHB throughout the exercise and recovery. Figure 3 is her resting ECG showing CHB with isorhythmic AV dissociation mimicking 2:1 block, atrial rate being around 80/minute, and ventricular rate being around 40/minute.

Her hospital course was uneventful, and the patient was discharged with an outpatient cardiology appointment. She was also scheduled to receive a loop recorder implantation.

## Discussion

CHB occurs when auricular and ventricular contractions are not communicated to each other beating at their own paces to result in a negative effect in cardiac function. CHB may occur in AV node, intra-Hisian, or infra-Hisian sites. Intranodal or intra-Hisian blocks almost always feature escape rhythms with narrow QRS complexes in the mean while infra-Hisian block often presents with wide QRS complex escapes.^1,2^

Our patient had CHB with narrow complex junctional escapes as shown in Figure 1. The escape rhythm responded well to sympathetic stimulation (exercise), suggesting that the level of block was intra-nodal or intra-Hisian with escapes from His-bundle. His-bundle escape rhythms are known to have narrow QRS complexes with an escape rate of 45 to 60 beats per minute. They are also responsive to alterations in autonomic tone such as physical exertion or pharmacological manipulation by atropine.^3^ As His-bundle escape rhythms are chronotropically competent to maintain adequate ventricular rate, those patients often remain asymptomatic. One should always consider the diagnosis of CCHB at the top of differentials tree when it comes to a young patient without past medical history presenting with CHB. Reversible and acquired etiologies of CHB need to be ruled out before committing to the diagnosis of congenital one. Comprehensive laboratory testing in our patient excluded Lyme carditis, electrolytes imbalance, or autoimmune diseases. Although cardiac sarcoidosis often presents with CHB, one of its characteristic ECG features includes wide QRS complexes owing to granulomatous infiltration of the conduction system.^4^ Therefore, CHB is most probably congenital in our case.

Most cases of CCHB are immune-mediated and presumed to be related to maternal anti-Ro/SSA and/or anti-La/SSB antibodies that enter the fetal circulation during gestation to result in fibrous degeneration of AV node and conduction system.^5-8^ CCHB may be isolated or associated with other structural heart diseases. One of the common complications of isolated CCHB is progressive enlargement of left ventricle leading to dilated cardiomyopathy even in asymptomatic patients. In a review of a multicenter retrospective study of 149 CCHB patients with CCHB, PM therapy may result in a decreased stress on the left ventricular over the time and may also benefit hemodynamically from prophylactic pacing. In the same study, most patients who received PM were found to have a decrease in their heart size during their follow-ups with echography.^9^ Data from few other studies also supported the aforementioned theory when considering prophylactic PM implantation.^10,11^ Strongly, PM therapy needs to be considered in asymptomatic patients with CCHB when there is average heart rate less than 50 beats per minute beyond the first year of life or less than 55 beats per minute in infants, presence of wide QRS complex escapes, ventricular dysfunction, prolonged QTc, or complex ventricular ectopy.^12-14^ Similar recommendations are also mentioned in current guidelines from the American Heart Association for PM therapy.^15,16^ Most of the asymptomatic patients with CCHB will eventually become symptomatic and require a PM at some point in their life.^17,18^ The only question is when would be the ideal time to implant a PM for those individuals who do not meet the above-mentioned criteria, since PM therapy itself also carries a significant rate of complications such as thrombosis, lead fractures, and so on, which may occur in up to 25% of cases.^11^ To the present day, the question remains unanswered.

There are some other diagnostic pearls worth to learn from ECGs in our case. Figure 3 appears to be a heart block with 2:1 pattern, which is mostly seen in second-degree AV block either Morbitz type I or type II. Given the fact that the patient has CCHB, the most likely diagnosis for Figure 3 is CHB with isorhythmic AV dissociation. It occurs when the rates of independent atrial and ventricular PMs approximate to each other or in an integral ratio, and one can be easily deceived as P waves are being conducted to ventricles although there is no actual synchronization between atrial and ventricular contractions.^19-22^ In addition, all the rhythms in Figures 1 to 3 also reveal an interesting phenomenon known as ventriculophasic sinus arrhythmia where PP intervals containing the QRS complexes are slightly shorter than the PP intervals without QRS complexes. The positive chronotropic effect of the ventricular systole resulting in the relative shortening of PP interval is probably derived from the traction of the contracting ventricle on the right atrium.^23^

## Conclusion

Treatment of asymptomatic CCHB with narrow complex escape rhythm is challenging. Those patients are often very young, and implanting a permanent PM is not always an easy decision. The likelihood of renewing multiple generators, potential of developing infections, and vascular complications sometimes outweigh the benefits of early intervention, and the ideal time for implanting a PM in those patients still remains a subject for further investigation. Nevertheless, the select group will benefit from close follow-ups, annual echocardiography, and rhythm monitoring by a loop recorder when they opt for a conservative approach without PM therapy.
